# The causal association between serum metabolites and lung cancer based on multivariate Mendelian randomization

**DOI:** 10.1097/MD.0000000000037085

**Published:** 2024-02-16

**Authors:** Tao Sun, Xiaoyang Chen, Hui Yan, Jun Liu

**Affiliations:** a Department of Hematology and Oncology Laboratory, The Affiliated Shaoyang Hospital, Hengyang Medical School, University of South China; b Department of Scientific Research and Teaching, The Affiliated Shaoyang Hospital, Hengyang Medical School, University of South China; c Department of Scientific Research, The First Affiliated Hospital of Shaoyang University, Shaoyang, Hunan Province, China.

**Keywords:** lung adenocarcinoma, Mendelian randomization, non-small cell lung cancer, serum metabolites, squamous lung cancer

## Abstract

This study seeks to understand the causal association between serum metabolites and different lung cancer types, an area yet to be extensively studied. We Used a two-sample Mendelian randomization (TSMR) approach, utilizing 486 blood metabolites as exposures and 3 distinct lung cancer types genome-wide association studies datasets as outcomes. We employed inverse variance weighting, MR-Egger, weighted median, simple mode, and weighted mode to estimate causal effects. We performed sensitivity analyses using Cochran Q test, MR-Egger intercept test, and MR-pleiotropy residual sum and outlier (MR-PRESSO). Linkage disequilibrium score (LDSC) analysis was conducted on the selected metabolites, and common confounding single nucleotide polymorphisms were eliminated using the human genotype-phenotype association Database. Metabolic pathway analysis was performed with MetaboAnalyst 5.0 software. Subsequently, a multivariate Mendelian randomization analysis was conducted to ascertain independent risk exposures. Our findings suggest independent risk factors for specific types of lung cancer: 7-methylxanthine and isoleucine for lung adenocarcinoma, cysteine and 1-arachidonoylglycerophosphocholine are identified as independent protective and risk factors for squamous lung cancer. Undecanoate (11:0) with Linoleate (18:2n6) showed a protective effect for small cell lung cancer. Additionally, 11 metabolic pathways were associated with lung cancer. This novel perspective offers a multidimensional understanding of lung cancer phenotypes, providing valuable guidance for identifying and screening of diverse lung cancer phenotypes.

## 1. Introduction

Lung cancer surpasses other cancer subtypes in terms of global cancer mortality, ranking first and constituting 18% of all global cancer deaths.^[1]^ Consequently, it stands as the second most prevalent cancer with the second highest incidence and the highest mortality rate worldwide.^[2]^ Lung cancer encompasses Non-small cell lung cancer (NSCLC) and small cell lung cancer (SCLC), with NSCLC accounting for over 85% of all lung cancer cases, predominantly comprising adenocarcinoma and squamous carcinoma.^[3,4]^ The absence of notable symptoms in early-stage lung cancer patients often leads to a delayed diagnosis, resulting in a considerable number of cases already advancing to intermediate and advanced stages.^[5,6]^ Consequently, the implementation of early screening measures for lung cancer becomes imperative in order to enhance the prognosis and extend the survival rate of individuals affected by this disease.

The alteration in metabolite levels is regarded as the primary reaction of biological systems to biological occurrences, capable of magnifying minor variations in genes and proteins and providing a more direct, precise, and sensitive reflection of the physiopathological condition of natural systems.^[7,8]^ Metabolomics, an integral facet of systems biology, strives to quantitatively assess all metabolites within biological systems in a comprehensive manner, elucidate the metabolic reprogramming of biological systems in response to inherent disturbances or extraneous stimuli, and uncover the associated metabolic regulatory mechanisms.^[9–11]^ Blood metabolomics has been instrumental in studying a range of malignant tumors, such as colorectal, breast, endometrial, and others.^[12–15]^ Lung cancer, being a significant subtype of cancer with high incidence and mortality rates, has benefited from the emergence and utilization of metabolomics.^[16,17]^ This has contributed to a more comprehensive comprehension of lung cancer development, offering novel perspectives and ideas for clinical diagnosis and treatment. While certain metabolites have been observed to have associations with lung cancer in population-based cohort studies, further research is needed to fully understand their role, the systematic evaluation of the impact of metabolites on lung cancer remains incomplete. Moreover, the utilization of observational studies to ascertain and establish potential causal associations between blood metabolites and lung cancer is challenging due to the presence of confounding factors that cannot be avoided.

Mendelian randomization analysis is an innovative and robust epidemiological approach that employs genetic variation as an unadulterated instrumental variable to investigate the causal association between disease exposure and clinical outcomes.^[18]^ By virtue of genotypes being established at conception and being less prone to confounding, MR analysis offers unbiased estimations.^[19,20]^ When we use the TSMR method, to avoid sample overlap, we can select 2 independent sample genome-wide association studies (GWAS) data from the same population, including the association results of genetic variation with risk factors and outcomes. The data of genetic variation-exposure factors and genetic variation-outcome variables were collected from 2 independent samples of the same population, such as the GWAS study of exposure factors and the GWAS study of outcome variables. In other words, the dataset has 2 sources: the association between genetic variation and endogenous variables (variant-exposure associations) is estimated in the first dataset, and the association between genetic variation and resulting variables (variant-outcome associations) is estimated in another dataset. In TSMR, exposure and outcome data need to come from the same population, but not necessarily from the same set of samples. TSMR is especially useful when exposing data and resulting data come from different sources or datasets. Capitalizing on this significant advantage, TSMR has gained considerable popularity in the last decade for inferring causal relationships pertaining to disease-related risk exposures, utilizing readily accessible GWAS summary statistics.^[19,21]^ The current study employed a TSMR approach to investigate the causal association between 486 blood metabolites and the susceptibility to various phenotypes of lung cancer, namely lung adenocarcinoma, squamous carcinoma, and SCLC. This research endeavor aimed to enhance our comprehension of the underlying mechanisms involved in the development of lung cancer, considering the uncertain causal link between blood metabolites and different lung cancer phenotypes. This study primarily aimed to examine the causal effects of human serum metabolites on lung adenocarcinoma, squamous lung cancer, and NSCLC. Additionally, it sought to identify shared metabolites that exert a causal effect on various lung cancer phenotypes, as well as to identify metabolic pathways that potentially contribute to the development of lung cancer across different phenotypes.

## 2. Materials and methods

### 2.1. Study design

**Figure 1.**
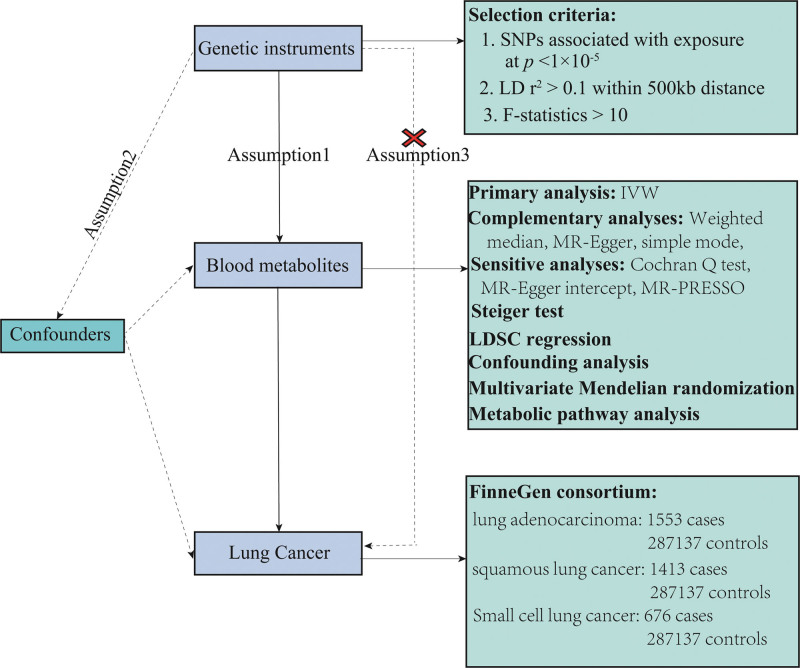
Flowchart of the study design.

This study employed a TSMR approach to evaluate the causal relationship between serum metabolites and the risk of 3 phenotypes of lung cancer. The MR analysis was guided by 3 primary hypotheses: the strong association between single nucleotide polymorphisms (SNPs) and exposure (blood metabolites), the absence of an association between SNPs and confounding factors, and the exclusive influence of SNPs on the outcome (lung cancer) through exposure (blood metabolites), indicating the absence of genetic pleiotropy.^[22,23]^ All MR analyses in this study were conducted using the R software version 4.2.1, employing the 2-Sample MR and MR-PRESSO packages.^[24]^ R language packages, such as ggplot2 and forest plot package, were applied to plot scatter plots and forest plots. The flow chart of this study is shown in Figure 1.

### 2.2. Data sources

We downloaded the most comprehensive pooled association statistics from the Human Metabolic Genetics Study,^[25,26]^ which were publicly accessible in the article published by Shin^[25]^ (website: https://pubmed.ncbi.nlm.nih.gov/24816252/). The study encompassed a total of 7824 individuals of European descent, with 1768 participants from the German KORA F4 study and 6056 participants from the UK Twin Study. The MS (Metabolism) platform was utilized to measure 486 metabolites, which were subsequently analyzed through GWAS using the HapMap2-based interpolated genotype dataset. After excluding a total of 177 metabolites with unknown identities, a total of 309 metabolites with known identities were successfully classified into 60 subclasses and 8 major groups.^[27]^ These major groups encompassed amino acids, carbohydrates, cofactors and vitamins, energy-related metabolites, lipids, nucleotides, peptides, and xenobiotic metabolism. For further details, please refer to Supplementary Table S1, Supplemental Digital Content, http://links.lww.com/MD/L401.

The FinnGen study is a distinctive endeavor that integrates genomic data with digital healthcare records of individuals aged 18 years and older residing in Finland.^[28]^ This comprehensive resource encompasses prospective epidemiological cohorts, disease-specific cohorts, and samples from hospital biobanks.^[29]^ Further details regarding this study have been provided elsewhere and are accessible through the official website (https://www.finngen.fi/fi). The GWAS summary data for lung adenocarcinoma, squamous lung cancer, and NSCLC were obtained from the Finngen Catalog (https://finngen.gitbook.io/documentation/). The sample size for lung adenocarcinoma was 288,690 individuals of Finnish descent, with a mean age at onset of 69.42 years. For squamous lung cancer, the sample size was 288,550 individuals, with a mean age at onset of 71.31 years. The full sample size for NSCLC included 287,813 individuals of Finnish descent, with 288,550 samples available and a mean age at onset of 70.36 years (Supplementary Table S2, Supplemental Digital Content, http://links.lww.com/MD/L405).

### 2.3. IVs selection

We first identified IVs associated with blood metabolites from multiple perspectives through stringent screening conditions. Given the small number of metabolite-associated SNPs, we relaxed the significance threshold *P* < 1 × 10^−5^ to filter out metabolite-associated SNPs.^[30,31]^ We then clustered SNPs by removing linkage disequilibrium (LD = 500 kb, *R*^2^ > 0.1). This criterion has been widely used in previous studies.^[12,32]^ To eliminate bias caused by poor instrumentation, we calculated the *R*^2^ and *F*-statistics for each SNP. The formula is *F* = *R*^2^(*n*-*k*-1)/(1-*R*^2^)*k*, where *n* represents the sample size and *k* represents the number of SNPs. Generally, an *F*-statistic above 10 is a typical threshold for selecting instrumental solid variables. *F*-statistics <10 for each metabolite will be filtered out.^[24,33]^

### 2.4. Statistical and sensitivity analyses

The primary assessment of the causal impact of blood metabolites on lung cancer was conducted using random-effects inverse variance weighting (IVW). The IVW estimates, derived from a pooled analysis of the Wald ratios of all genetic variants, were employed to screen for blood metabolites with a causal effect on the 3 phenotypes of lung cancer. The assumption underlying IVW is the absence of horizontal pleiotropy across all SNPs, making it the most accurate method for evaluating causal effects. In order to enhance the reliability of the findings, we employed 4 supplementary approaches to assess metabolites with notable estimates (*P* < .05 derived by IVW). MR-Egger, the weighted median method, the simple mode, and the weighted mode were designated as complementary analyses. These complementary analytical methods make MR results more reliable. Sensitivity analysis assumes crucial significance as it examines the presence of horizontal pleiotropy and heterogeneity, which can significantly compromise MR estimates. Horizontal pleiotropy arises when instrumental variables exert an influence on the outcomes via pathways unrelated to the focal exposure. Consequently, a series of tests were conducted in order to generate reliable estimates. This study employed 3 methodologies, namely the Cochran Q test, the MR-Egger intercept test, and the MR-PRESSO, to identify and address heterogeneity and multicollinearity. A *P* value of <.05 from the Cochran Q test indicated the presence of heterogeneity in the results.^[24]^ The MR-Egger intercept was computed to examine potential bias resulting from directional pleiotropy and null instrumental variables.^[34,35]^ Lastly, MR-PRESSO was utilized to further assess the existence of heterogeneous SNPs.^[36,37]^

In summary, a thorough screening of blood metabolites with potential causal effects on 3 different phenotypes of lung cancer was conducted using multiple criteria: The primary analysis yielded a *P* value that is statistically significant (IVW-derived *P* < .05), the 5 MR methods consistently demonstrate the same direction, there is no heterogeneity and horizontal pleiotropy in MR results, and The MR estimate is not heavily corrupted by individual SNPs. Furthermore, to evaluate the statistical power of these estimates, we utilized an online website (https://shiny.cnsgenomics.com/mRnd/). Specifically, this tool employs asymptotic theory to calculate power values for detecting causal effects of Ivs.^[38,39]^

### 2.5. Genetic correlation and direction validation analysis

Multiple studies have substantiated the occurrence of false positives in MR when genetic correlations exist between traits. Despite the exclusion of SNPs linked to lung cancer during the instrumental variable selection process, it is plausible that combinations of SNPs lacking significant association with lung cancer could still contribute to the genetic susceptibility of this disease. Consequently, in order to examine the potential impact of shared genetic structure on the observed causality, the genetic associations between the identified metabolites and 3 distinct lung cancer phenotypes were evaluated using LDSC.^[40]^

Furthermore, we conducted a validation process to assess the potential influence of reverse causality on the observed causal associations, employing the Steiger test.^[41]^ By means of the Steiger test, we ascertained whether the included SNPs accounted for a greater proportion of the variability in lung cancer compared to the identified metabolites. In instances where combinations of SNPs were found to exert a more substantial impact on the genetic susceptibility to lung cancer than metabolites (Steiger *P* > .05), it implies a potential bias in the direction of causal inference.

### 2.6. Confounding analyses

In order to evaluate the horizontal pleiotropy of the MR results, a series of sensitivity analyses were conducted to identify any SNPs that violated the assumptions of MR. However, it is possible that a small residual number of confounding SNPs may still exist. To assess the association of each SNP with established common risk factors for lung cancer, including smoking,^[42]^ alcohol consumption,^[43]^ BMI,^[44]^ hypertension,^[45]^ diabetes,^[46]^ and high cholesterol.^[47]^ The Phenoscanner V2 website (http://www.phenoscanner.medschl.cam.ac.uk/) was utilized. Any SNPs found to be significantly associated with the aforementioned confounders (*P* < 1 × 10^−5^) were considered, and MR analysis would be re-performed after removing these SNPs to verify the reliability of the results.

### 2.7. Multivariate MR analysis

To avoid violating MR hypotheses 2 and 3 (Fig. 1), The analysis of MR should ensure that genetic variants are linked to a singular risk factor. Nevertheless, in practice, certain genetic variants may be associated with multiple risk factors, a phenomenon referred to as multiple effects. In such instances, multivariable Mendelian randomization (MVMR) can account for the interplay between genetic variants and exposure factors by incorporating multiple exposure factors that may interact with 1 another.^[48]^ In summary, univariate MR evaluates the collective impact of an exposure on an outcome, while MVMR examines the individual effects of each exposure (independently of any other) on an outcome. In this study, we conducted MVMR analysis on the identified metabolites to account for their potential interactions. MVMR was implemented using both the IVW and MR-PRESSO methods. The IVW method in multivariate Mendelian randomization entails regressing all exposed SNPs against the outcome variable and assigning weights based on the inverse variance of the outcome.^[49]^ MR-PRESSO, on the other hand, identifies and removes outliers to address the multidirectionality of instrumental variables.^[37]^ Influences that exhibited significant differences (*P* < 1 × 10^−5^) according to both IVW and MR-PRESSO were considered as independent factors.

### 2.8. Metabolic pathway analysis

In order to elucidate the biological mechanisms underlying the impact of blood metabolites on the 3 distinct phenotypes of lung cancer, we conducted a metabolic pathway analysis utilizing MetaboAnalyst 5.0 (https://www.metaboanalyst.ca/).^[50]^ This analysis aimed to investigate the potential pathogenesis associated with the 3 different phenotypes of lung cancer.

### 2.9. Ethics statement

This MR analysis utilized publicly available summary statistics from GWAS. The participants in the original studies were granted written informed consent, as approved by the Institutional Review Board ethics committees. Consequently, no further ethical approval or informed consent was deemed necessary.

## 3. Results

### 3.1. Instrumental variables

We conducted two-sample MR analyses using GWAS summary data from 3 distinct lung cancer phenotypes to evaluate the causal relationships between serum metabolites and lung cancer. A total of 486 metabolites were examined, with IVs derived from 2 to 301 SNPs for each metabolite. The metabolite M32322 had the fewest IVs (2 SNPs), while M33178 had the highest number of IVs (301 SNPs) (Supplementary Table S3, Supplemental Digital Content, http://links.lww.com/MD/L408). Additionally, the *F*-statistics for these IVs ranged from 17.45 to 1431.87, indicating that all IVs were sufficiently valid (*F*-statistic > 10) for the MR analysis of the 486 metabolites.

### 3.2. Results of MR analysis

**Figure 2.**
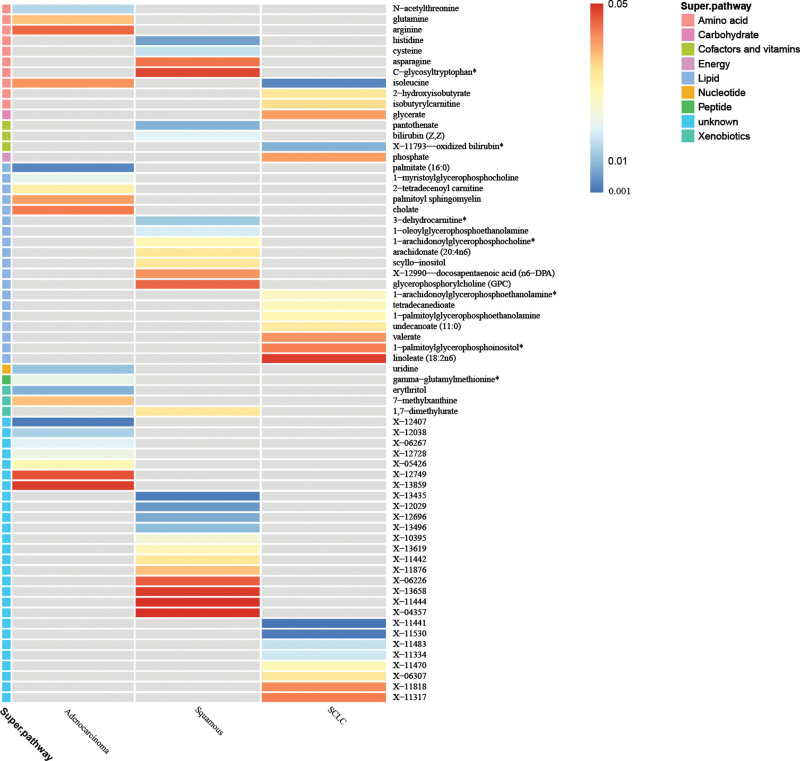
Mendelian randomized associations of 66 serum metabolites (*P*_IVW_ < .05) with risk of 3 lung cancer phenotypes.

In the initial investigation, we have identified a total of 20 metabolites that may be associated with lung adenocarcinoma. Among these, 13 metabolites have established chemical properties, while the remaining 7 have properties that are yet to be determined. Furthermore, our analysis has revealed 25 metabolites implicated in the pathogenesis of lung squamous carcinoma, with 13 having known chemical properties and 12 being unknown. Additionally, we have identified 21 metabolites that potentially exert pathogenic effects on NSCLC. Specifically, 13 of these metabolites have established chemical properties, while the remaining 8 have properties that are currently unknown (Fig. 2). As shown, the known serum metabolites were assigned to amino acids, carbohydrates, dipeptides, lipids, nucleotides, peptides, and probiotics.

### 3.3. Results of sensitivity analysis

**Figure 3.**
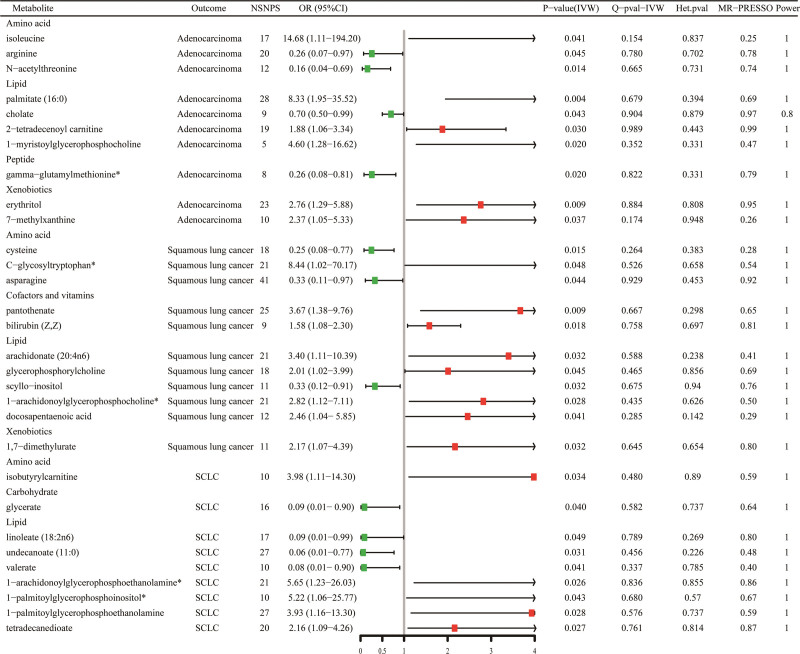
Forest plot of causal relationship between blood metabolites and 3 different phenotypes of lung cancer derived from MR analysis and sensitivity analysis.

**Figure 4.**
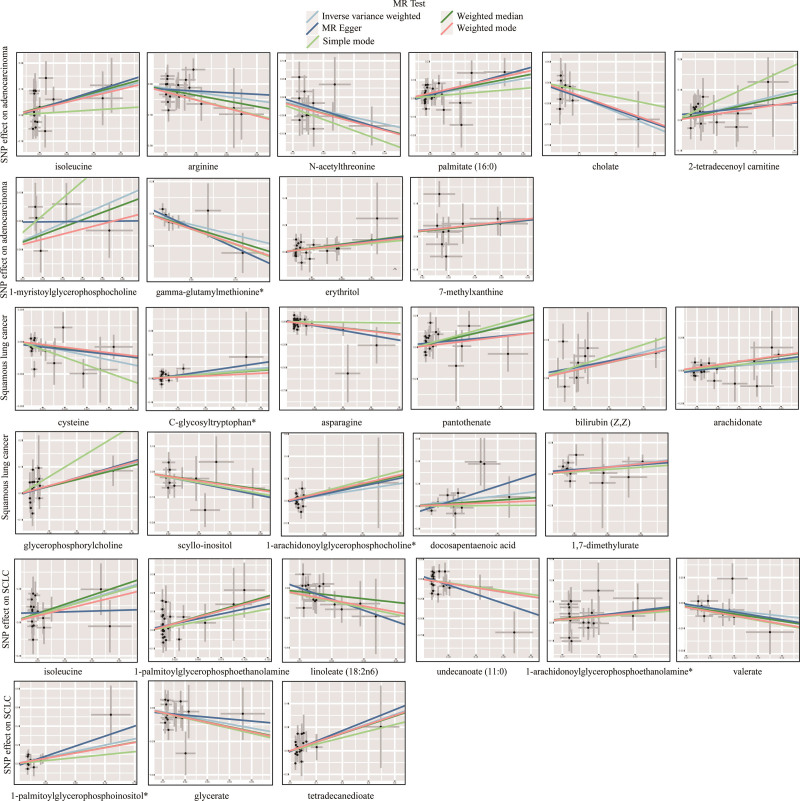
Scatterplot of 5 MR methods with consistent orientation.

Sensitivity analyses were conducted to evaluate potential bias in the assumptions of MR for the significant estimates (*P*_IVW_ < .05) identified. Cochran Q-generated *p*-values indicated the absence of heterogeneity. Furthermore, the MR-Egger intercept term indicated a low risk of horizontal pleiotropy, as shown in Supplementary Table S4, Supplemental Digital Content, http://links.lww.com/MD/L410. To mitigate the polytropic effect, the MR-PRESSO method was employed to identify SNP outliers. It was found that all *P*_MR-PRESSO_ were >.05, and no aberrant SNPs were found. The results of the MR-PRESSO analyses are shown in Supplementary Table S5, Supplemental Digital Content, http://links.lww.com/MD/L412. Through the integration of complementary methodologies and sensitivity analyses, along with the exclusion of unidentified metabolites, a total of 30 metabolites (10 lung adenocarcinomas, 11 squamous lung carcinomas, and 9 non-small cell lung carcinomas) that fulfilled the rigorous screening criteria were identified as potential candidates (Fig. 3). Specifically, isoleucine (OR_IVW_ = 14.68; 95% CI: 1.11–194.2; *P*_IVW_ = .041), C-glycosyltryptophan* (OR_IVW_ = 8.44; 95% CI: 1.02–70.17, *P*_IVW_ = .048), 1-arachidonoylglycerophosphoethanolamine* (OR_IVW_ = 5.65; 95% CI: 1.23–26.03; *P*_IVW_ = .026) were the most hazardous metabolites for lung adenocarcinoma, squamous lung cancer, and NSCLC, respectively. In contrast, N-acetylthreonine (OR_IVW_ = 0.16; 95% CI: 0.04–0.69; *P*_IVW_ = .014), asparagine (OR_IVW_ = 0.33; 95% CI: 0.11–0.97; *P*_IVW_ = .044), undecanoate (11:0) (OR_IVW_ = 0.06; 95% CI: 0.01–0. 77; *P*_IVW_ = .031) were the factors with the highest protective value for lung adenocarcinoma, squamous lung cancer, and NSCLC respectively. The statistical efficacy of all estimates was > 0.8, except for 5 metabolites, for which power values could not be calculated (Supplementary Table S6, Supplemental Digital Content, http://links.lww.com/MD/L415). Figure 4 demonstrates the scatterplot of these 30 candidate metabolites.

### 3.4. Assessment of genetic correlation and directionality

In order to mitigate potential confounding factors arising from the alignment of exposure and outcomes, we employed LDSC to investigate the genetic correlation between the screened metabolites and the 3 distinct phenotypic lung cancers. Our analysis revealed no discernible genetic correlation between the aforementioned candidate metabolites and the 3 phenotypic lung cancers, indicating that the estimates derived from Mendelian randomization were not influenced by shared genetic elements (Supplementary Table S7, Supplemental Digital Content, http://links.lww.com/MD/L416). The MR estimates remained unaffected by the presence of shared genetic elements. Additionally, a Steiger test was conducted to confirm the direction of the impact of metabolites on lung cancer. The Steiger *P* value provided evidence that the observed causal relationship was not influenced by reverse causality (Supplementary Table S8, Supplemental Digital Content, http://links.lww.com/MD/L417).

### 3.5. Confounding analysis

We conducted an analysis of metabolite IVs using the Phenoscanner V2 website (http://www.phenoscanner.medschl.cam.ac.uk/) to evaluate the potential association between each SNP and established risk factors for lung cancer, such as smoking, alcohol consumption, hypertension, diabetes, cholesterol, and body mass index (BMI). Our investigation identified 18 SNPs associated with metabolites that were also linked to the aforementioned confounding factors (Supplementary Table S9, Supplemental Digital Content, http://links.lww.com/MD/L422). Subsequently, we proceeded to repeat the MR analysis after excluding these SNPs. The results showed that 5 metabolites were no longer significantly causally associated with lung cancer: lung adenocarcinoma, erythritol (OR_IVW_ = 2.20, 95% CI = 0.77–6.30, *P* = .143), lung squamous carcinoma, arachidonate (20:4n6) (OR_IVW_ = 0.71, 95% CI = 0.14–3.63, *P* = .685), glycerophosphorylcholine (OR_IVW_ = 1.66, 95% CI = 0.81–3.38, *P* = .163), bilirubin (Z, Z) (OR_IVW_ = 1.79, 95% CI = 0.92–3.50, *P* = .087), docosapentaenoic acid (OR_IVW_ = 1.59, 95% CI = 0.63–4.00, *P* = .326) (Supplementary Table S10, Supplemental Digital Content, http://links.lww.com/MD/L424). We removed these 5 metabolites from subsequent multivariate Mendelian randomization analyses.

### 3.6. Multivariate Mendelian randomization analysis

**Figure 5.**
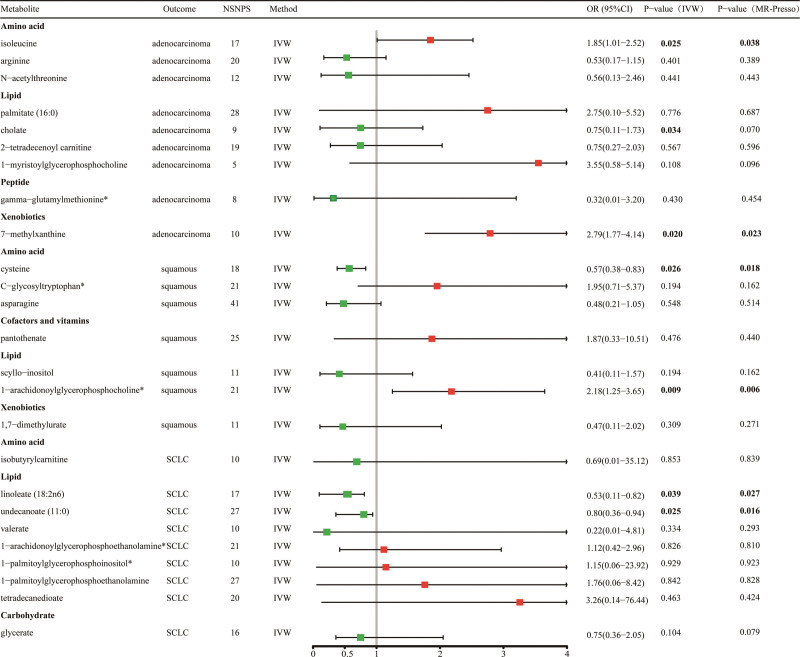
Forest plot of multivariate MR analysis results.

We ultimately incorporated a total of 25 metabolites for the purpose of conducting a multivariate Mendelian randomization analysis, including lung adenocarcinoma (isoleucine, palmitate [16:0], arginine, cholate, gamma-glutamylmethionine*, N-acetylthreonine, 7-methylxanthine, 2-tetradecenoyl carnitine, 1-myristoylglycerophosphocholine,), lung squamous carcinoma (pantothenate, cysteine, scyllo-inositol, C-glycosyltryptophan*, 1-arachidonoylglycerophosphocholine*, asparagine, 1,7-dimethylurate), NSCLC (linoleate [18:2n6], glycerate undecanoate [11:0], isobutyrylcarnitine, valerate, 1-arachidonoylglycerophosphoethanolamine*, 1-palmitoylglycerophosphoinositol*, 1-palmitoylglycerophosphoethanolamine, tetradecanedioate). After accounting for metabolite interactions, the MVMR estimates derived from the IVW and MR-PRESSO methodologies revealed that isoleucine (OR_IVW_ = 1.85, 95% CI = 1.01–2.52, *P*_IVW_ = 0.025, *P*_MR-Presso_ = .038) and 7-methylxanthine (OR_IVW_ = 2.79, 95% CI = 1.77–4.14, *P*_IVW_ = 0.020, *P*_MR-Presso_ = .023) possess a direct impact on lung adenocarcinoma. Similarly, cysteine (OR_IVW_ = 0.57, 95% CI = 0.38–0.83, *P*_IVW_ = .026, *P*_MR-Presso_ = .018) and 1-arachidonoylglycerophosphocholine* (OR_IVW_ = 2.18, 95% CI = 1.25–3.65, *P*_IVW_ = .009, *P*_MR-Presso_ = .006) were found to directly influence squamous lung cancer. Furthermore, linoleate (18:2n6) (OR_IVW_ = 0.53, 95% CI = 0.11–0.82, *P*_IVW_ = .039, *P*_MR-Presso_ = .027) and undecanoate (11:0) (OR_IVW_ = 0.80, 95% CI = 0.36–0.94, *P*_IVW_ = .025, *P*_MR-Presso_ = .016) were observed to independently affect non-small-cell lung cancers (Fig. 5).

### 3.7. Results of metabolic pathway analysis

**Figure 6.**
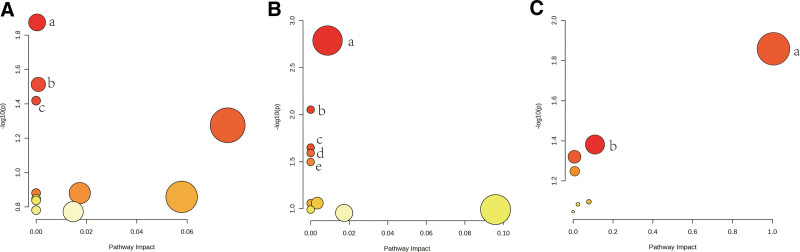
Eleven metabolic pathways that may be involved in the pathogenesis of lung cancer. (A) a, Aminoacyl-tRNA biosynthesis. b, Valine, leucine and isoleucine biosynthesis. c, Caffeine metabolism. (B) a, Pantothenate and CoA biosynthesis. b, Aminoacyl-tRNA biosynthesis. c, Thiamine metabolism. d, Taurine and hypotaurine metabolism. e, Caffeine metabolism. (C) a, Linoleic acid metabolism. b, Glycerolipid metabolism.

Eleven significant metabolic pathways were identified in the 3 lung cancer phenotypes through metabolic pathway analysis (Fig. 6). The findings indicate that the “aminoacyl-tRNA biosynthesis” (*P* = .013), “valine, leucine, and isoleucine biosynthesis” (*P* = .031), and “caffeine metabolism” (*P* = .038) pathways are associated with the pathogenesis of lung adenocarcinoma. On the other hand, the “pantothenate and CoA biosynthesis” (*P* = .001), “aminoacyl-tRNA biosynthesis” (*P* = .009), and “thiamine metabolism” (*P* = .022), “Taurine and hypotaurine metabolism” (*P* = .026) and “caffeine metabolism” (*P* = .032) pathways are believed to be associated with squamous lung cancer. The pathogenesis of NSCLC was found to be associated with the “linoleic acid metabolism” (*P* = .013) and “glycerolipid metabolism” (*P* = .041) pathways. Additionally, our analysis revealed that certain metabolic pathways, such as “Aminoacyl-tRNA biosynthesis” and “Caffeine metabolism,” were shared by both lung adenocarcinoma and squamous lung cancer phenotypes (Supplementary Table S11, Supplemental Digital Content, http://links.lww.com/MD/L431).

## 4. Discussion

This study employed a comprehensive approach by utilizing large-scale GWAS data from publicly available databases to investigate the causal association between 486 blood metabolites and the susceptibility to lung adenocarcinoma, squamous lung cancer, and SCLC. The analysis was conducted using an unbiased two-sample Mendelian randomization methodology. Following rigorous quality control measures and multivariate Mendelian randomization analysis, the findings indicated that 7-methylxanthine and Isoleucine were identified as independent risk factors for lung adenocarcinoma, while cysteine and 1-arachidonoylglycerophosphocholine exhibited roles as protective factors and risk factors for squamous lung cancer respectively. Undecanoate and linoleate were found to be independent protective factors for SCLC. Following this, we have identified eleven metabolic pathways that potentially play a role in the biological mechanisms underlying the development of lung cancer. To the best of our knowledge, this is the initial study utilizing the most comprehensive blood metabolite GWAS data to investigate causal associations with 3 distinct lung cancer phenotypes, employing a combination of metabolic pathways and multivariate Mendelian randomization analysis.

The results indicate a potential correlation between the presence of 7-methylxanthine and Isoleucine and an elevated likelihood of developing lung adenocarcinoma. 7-Methylxanthine, a metabolite derived from caffeine (1,3,7-trimethylxanthine), has been extensively investigated for its impact on various ailments such as neurodegenerative disorders, respiratory conditions, diabetes, and cancer. Notably, in a study involving mice with colon cancer, the response to 7-methylxanthine was observed to be significantly enhanced. However, the administration of a combination of *Astragalus membranaceus* and *Curcuma wenyujin* (AC) resulted in a reduction in levels of 7-methylxanthine. This finding implies that AC possesses the ability to regulate alterations in 7-methylxanthine through modulation of the caffeine metabolic pathway. Consequently, it is plausible to suggest that AC may impede the proliferation and dissemination of colon cancer by influencing the metabolism of 7-methylxanthine.^[51]^ Nevertheless, there is a dearth of research investigating the involvement of 7-methylxanthine in the context of lung cancer. Recent research indicates a potential association between Isoleucine and the emergence and advancement of lung cancer. An examination conducted on individuals afflicted with lung cancer revealed notably elevated concentrations of Isoleucine within tumor tissue compared to healthy tissue. Furthermore, a positive correlation was observed between Isoleucine levels and both tumor size and malignancy, implying a potentially significant role of Isoleucine in the development of lung cancer.^[52]^ Additionally, laboratory investigations have demonstrated that Isoleucine fosters the proliferation and invasive capabilities of tumor cells,^[53]^ aligning with the overall findings of this study. However, it is imperative to acknowledge that a limited number of studies have been undertaken to investigate the correlation between Isoleucine and lung cancer, necessitating further research to validate this association. Consequently, future investigations should also delve into elucidating the mechanism by which Isoleucine operates and how this discovery can be effectively employed in anti-lung cancer therapeutic approaches.

In the study conducted by Brown et al, it was discovered that the concentration of cysteine in fecal water extracts obtained from individuals diagnosed with colorectal cancer exhibited a notable increase when compared to that of healthy subjects.^[54]^ This observation potentially signifies modifications in the production and functionality of mucus, which serves as the protective covering of the colorectal mucosa. Additionally, the authors of the study identified cysteine’s involvement in the synthesis of nitric oxide within the human body. Nitric oxide, a molecule with a dual nature, possesses both anticancer properties as well as the potential to facilitate tumor growth and metastasis.^[55]^ 1-arachidonoylglycerophosphocholine is a member of the lysoPC family, which plays a crucial role in tumor metastasis and growth during assimilation, metabolism and catabolism of tumor cells. It has been observed that the plasma levels of LysoPC are diminished in individuals with various tumors, and these levels are associated with clinical indicators such as inflammatory markers and weight loss.^[56]^ Nevertheless, further investigations are required to elucidate the precise interpretation of these findings, which will provide a comprehensive understanding of the mechanism involving cysteine and 1-arachidonoylglycerophosphocholine in squamous lung cancer.

The role of undecanoate in cancer development has received limited attention in research, with most studies focusing on its antifungal infection activity and therapeutic strategies. Undecanoate is a monocarboxylic acid that exhibits antifungal effects by inhibiting the production of extracellular keratinase, lipase, and several phospholipids in Trichophyton rubrum. Its antimicrobial mechanism primarily involves altering the cell membrane and cell wall structure of bacteria and fungi, leading to oxidative stress and metabolic disruption, ultimately resulting in the antimicrobial effect.^[57]^ Empirical investigations have demonstrated that linoleate possesses the ability to diminish serum LDL cholesterol levels, thereby potentially mitigating the risk of coronary heart disease. In an effort to consolidate the existing evidence pertaining to the correlation between linoleate and breast cancer risk.^[58]^ Yunping Zhou et al introduced a meta-analysis methodology. Their findings indicated a positive association between linoleate consumption levels and a decreased likelihood of developing breast cancer.^[59]^ These outcomes broadly align with our own research outcomes. Nevertheless, it is crucial to acknowledge that the validity of these associations may be influenced by potential confounding factors and inaccuracies in measurement. For instance, individuals who are exposed to high levels of linoleate may exhibit elevated levels of physical activity, reduced prevalence of overweight/obesity, and decreased consumption of alcohol and fat. Despite the fact that numerous studies have accounted for these variables and other potential confounding factors, there is still a potential for bias. However, our utilization of Mendelian randomization in investigating the causal association between linoleate and lung cancer successfully mitigated the influence of these confounders.

This study demonstrates innovation through the following means: Firstly, it explores the molecular mechanism by investigating the causal association between blood metabolites and the risk of 3 phenotypes of lung cancer, employing blood metabolites as the exposure factors. This approach is grounded in a robust theoretical foundation and holds significant clinical research significance. Secondly, the study employs rigorous quality control measures and analytical techniques, employing multiple models to evaluate the causal effect. As a result, the findings are both reliable and consistent. Thirdly, In contrast to prior Mendelian randomization investigations focusing on a solitary exposure factor, the present study encompassed a multitude of metabolites in the bloodstream, thereby presenting a substantial workload and analytical complexity. This study has several limitations that should be acknowledged. Firstly, the GWAS data utilized in this study were obtained exclusively from European populations, necessitating the need for future investigations to encompass diverse populations for a more comprehensive analysis. Secondly, despite employing the most extensive GWAS data available to date, subsequent studies should aim to expand the sample size further to ensure a more precise evaluation of the genetic influence of metabolites. In addition, although MR analysis provides valuable insights into etiology, it must be noted that the sample set and conclusions used in the study should be verified by rigorous RCT and basic research before being applied to the clinic.

## 5. Conclusion

We employed a two-sample Mendelian randomization methodology to investigate the causal link between 486 blood metabolites and 3 lung cancer phenotypes. While existing research has presented convincing evidence of metabolite involvement in the biological mechanisms of lung cancer, thereby potentially benefiting its treatment, the extent to which they contribute to early screening and prevention of lung cancer remains restricted due to the ambiguous causal association between the 2. Consequently, a pivotal MR study was undertaken with the objective of elucidating the causal association between blood metabolites and the 3 distinct phenotypes of lung cancer, as well as the metabolic pathways implicated. This study aims to offer valuable insights for the screening and therapeutic approaches targeting diverse lung cancer phenotypes.

## Acknowledgments

We want to acknowledge the participants and investigators of the FinnGen study. In addition, we would like to thank Home for Researchers for providing proofreading services for the paper (https://www.home-for-researchers.com).

## Author contribution

Data curation: Tao Sun, Xiaoyang Chen.

Formal analysis: Tao Sun.

Methodology: Tao Sun.

Writing – original draft: Tao Sun.

Software: Xiaoyang Chen.

Writing – review & editing: Xiaoyang Chen.

Project administration: Hui Yan.

Conceptualization: Jun Liu.

Supervision: Jun Liu.

## Supplementary Material






















